# Automated machine learning‐based model predicts postoperative delirium using readily extractable perioperative collected electronic data

**DOI:** 10.1111/cns.13758

**Published:** 2021-11-18

**Authors:** Xiao‐Yi Hu, He Liu, Xue Zhao, Xun Sun, Jian Zhou, Xing Gao, Hui‐Lian Guan, Yang Zhou, Qiu Zhao, Yuan Han, Jun‐Li Cao

**Affiliations:** ^1^ Department of Anesthesiology The Affiliated Hospital of Xuzhou Medical University Jiangsu Province Xuzhou City China; ^2^ Jiangsu Province Key Laboratory of Anesthesiology & NMPA Key Laboratory for Research and Evaluation of Narcotic and Psychotropic Drugs Xuzhou Medical University Jiangsu Province Xuzhou City China; ^3^ Department of Anesthesiology The Affiliated Huzhou Hospital Zhejiang University School of Medicine Huzhou Central Hospital Zhejiang Province Huzhou City China; ^4^ Department of Anesthesiology Changzhou First People's Hospital Changzhou Jiangsu China; ^5^ Department of Anesthesiology The First Affiliated Hospital of Bengbu Medical College Bengbu Anhui China; ^6^ Department of Anesthesiology Eye & ENT Hospital of Fudan University Shanghai China

**Keywords:** delirium, machine learning, model prediction, nomogram, postoperative

## Abstract

**Objective:**

Postoperative delirium (POD) is a common postoperative complication that is relevant to poor outcomes. Therefore, it is critical to find effective methods to identify patients with high risk of POD rapidly. Creating a fully automated score based on an automated machine‐learning algorithm may be a method to predict the incidence of POD quickly.

**Materials and methods:**

This is the secondary analysis of an observational study, including 531 surgical patients who underwent general anesthesia. The least absolute shrinkage and selection operator (LASSO) was used to screen essential features associated with POD. Finally, eight features (age, intraoperative blood loss, anesthesia duration, extubation time, intensive care unit [ICU] admission, mini‐mental state examination score [MMSE], Charlson comorbidity index [CCI], postoperative neutrophil‐to‐lymphocyte ratio [NLR]) were used to established models. Four models, logistic regression, random forest, extreme gradient boosted trees, and support vector machines, were built in a training set (70% of participants) and evaluated in the remaining testing sample (30% of participants). Multivariate logistic regression analysis was used to explore independent risk factors for POD further.

**Results:**

Model 1 (logistic regression model) was found to outperform other classifier models in testing data (area under the curve [AUC] of 80.44%, 95% confidence interval [CI] 72.24%–88.64%) and achieve the lowest Brier Score as well. These variables including age (OR = 1.054, 95%CI: 1.017~1.093), extubation time (OR = 1.027, 95%CI: 1.012~1.044), ICU admission (OR = 2.238, 95%CI: 1.313~3.793), MMSE (OR = 0.929, 95%CI: 0.876~0.984), CCI (OR = 1.197, 95%CI: 1.038~1.384), and postoperative NLR (OR = 1.029, 95%CI: 1.002~1.057) were independent risk factors for POD in this study.

**Conclusions:**

We have built and validated a high‐performing algorithm to demonstrate the extent to which patient risk changes of POD during the perioperative period, thus leading to a rational therapeutic choice.

## Graphical abstract

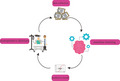

## 1 INTRODUCTION

Postoperative delirium (POD) is an acute fluctuating neurocognitive syndrome caused by reversible neuronal disruption due to an underlying systemic perturbation, which usually occurs a few hours to a few days after surgery and mainly manifests as a decline in consciousness, attention disorders, and thinking disorders.^1^ It has been reported that the incidence of POD in elderly surgical patients ranges from 10% to 70%.^2^, ^3^

Previous studies have demonstrated that early interventions can help reduce or even prevent POD,^4^ while many patients with POD can't be identified efficiently. In clinical settings, the diagnosis of POD is still mainly based on clinical observation.^5^ However, the type of hypoactive POD is about 71% and very hard to notice. Therefore, it is critical to find methods to identify patients with a high risk of POD rapidly.

In recent years, basic and clinical studies have found that many risk factors or biomarkers may affect the occurrence of POD.^6^, ^7^ For instance, many inflammatory markers investigated in scientific and clinical studies, such as CRP, were believed to be associated with POD.^8^, ^9^, ^10^ Therefore, disease prediction models conveniently screen high‐risk patients, and the nomogram could be easily used in clinical settings. However, some prediction models for POD were based on a single statistical method, which may be limited in predictive performance.^11^, ^12^ Recently, it has been reported that using machine‐learning techniques to establish various disease prediction models could improve the predictive performance of these models.^13^, ^14^

Thus, in the current study, we used machine‐learning technology to extract the clinical data of 531 surgical patients who underwent general anesthesia before and on the first day after surgery and established four predictive models of POD using different methods. Finally, we compared these models and created a model with optimal predictive performance, which can assist in diagnosing and identifying patients with a high risk of POD. Furthermore, to increase the availability of the optimal model, the optimal model was transformed into the form of a nomogram.

## 2 MATERIALS AND METHODS

### 2.1 Data source and extraction

The secondary analysis was based on an observational study (the Ethical Committee of the Affiliated Hospital of Xuzhou Medical University approved it, Certification No. XYFY2018‐KL091). The written informed consent was obtained from all subjects participating, a legal surrogate, or the parents in this trial. Inclusion criteria were as follows: non‐history of clear neurological disease; patients who underwent major noncardiac or non‐neurological surgery with general anesthesia; expected a hospital stay of ≥3 days; Exclusion criteria were as follows^15^: significant impairments of vision; hearing or motor skills; history of neurological disease; liver or kidney dysfunction (such as severe hepatitis, pyelonephritis); severe trauma or surgical history within one year; history of severe physical illness and alcoholism; mini‐mental state examination (MMSE) score < 17; refuse to sign informed consent.

### 2.2 Model endpoint definition

We built classification models to predict the in‐hospital incidence of POD as a binary outcome.

### 2.3 Delirium assessment

Delirium was assessed using rigorous methodologies. In this trial, CAM^16^ was applied to patients who could be communicated with. The CAM‐ICU^17^ was applied to patients admitted to the intensive care unit (ICU) and cannot be communicated with due to endotracheal intubation. We assessed for delirium 2 h after the surgery and then repeated the assessment twice a day for three days after the surgery in the morning, afternoon, or evening. There was at least 6 h interval between these two assessments.^18^ Additionally, evidence of delirium, including confusion, agitation, sedation, hallucinations, and delusions, was obtained from the nurses, families, and medical records. The evaluation of delirium was carried out by trained researchers who neither knew the patient's perioperative characteristics nor data entry and statistical analysis.

### 2.4 Model input features

Forty‐nine potential useful features including basic information such as age, sex, BMI, education degree; American society of anesthesiologists (ASA) degree; laboratory data obtained before surgery, such as serum sodium, potassium, creatinine, and blood cell counts; and surgery‐specific information such as the surgery type were collected.

Least absolute shrinkage and selection operator (LASSO) was used to select important features associated with POD. Finally, eight features (age, intraoperative blood loss, anesthesia duration, extubation time, ICU admission, mini‐mental state examination score [MMSE], Charlson comorbidity index [CCI], postoperative neutrophil‐to‐lymphocyte ratio [NLR]) were included to established models.

To achieve the highest predictive performance, four models were established, including logistic regression model (LR), random forest (RF), extreme gradient boosted trees (XGB) classifier, and support vector machine (SVM) classifier. Furthermore, in order to further explore the relationship between the above eight features and POD, multivariate logistic regression was used to confirm the independent risk factors for POD in this study.

### 2.5 Sample size and statistical analysis

For the two‐class prediction model, one of the sample size calculation methods proposed in the article is^19^ $n=\exp\left(\frac{‐0.508+0.259\ln(\varphi )+0.504\ln(P)‐\ln(MAPE)}{0.544}\right)$, where *φ* is the proportion of ending events (φ=0.23), *P* is the number of predictors (*P* = 8), *MAPE* is the average absolute error between the observed and true outcome probability (*MAPE* = 0.05). According to the above formula, the sample size of training set is calculated as at least 330. To achieve a sample size of at least 330 in the training set, we randomly split the total dataset (*n* = 531) into a training set (*n* = 400) and a testing set (*n* = 131) at a ratio of 7:3 in this study. Any patients who appeared in the testing set would be removed from the training set in case of information leakage.

All analyses were performed with R version 3.6.1. (R Development Core Team). The normal distribution of numeric variables was tested by the Shapiro‐Wilk test. Continuous variables with a normal distribution were expressed as the mean ± standard deviation (SD) and were compared using the independent‐sample *t*‐test. The Mann–Whitney *U* test presented continuous variables with a non‐normal distribution. Categorical data were presented as a number (%) and were analyzed using the chi‐square test or Fisher's exact probability test. The importance of each variable in the training datasets was assessed by LASSO regression analysis.

The selection of model hyperparameters used 10‐fold cross‐validation on training datasets. In 10‐fold cross‐validation, the datasets were divided into ten partitions, where nine‐tenths of the data were used to build the models, and the remaining one‐tenths were used as the testing datasets. This process was repeated such that each partition was used as testing datasets only once and training datasets nine times. Cross‐validation made ensures a better assessment of model performance by averaging metrics over multiple trials.

The role of missing data imputation is described as follows. If the missing value percentage is more significant than 20%, it will be excluded from the final completed dataset. If the rate of missing value is smaller than 20%, the random forest regression method would be used for imputation.

Discrimination and calibration were used to verify the predictive ability of the model. The AUROC expressed measurement of discrimination, and the Youden index (sensitivity + specificity − 1) was used to find the best critical value (cutoff value). The performance of models was evaluated by accuracy, sensitivity, specificity, recall, and precision. Model calibration was measured by Brier score and calibration curve. Brier score was the average squared distance between the predicted probability of the outcome and the true label, and the lower Brier score indicated the better performance of the model.

The LR and RF classifiers were implemented with glmnet package and randomForest package in R version 3.6.1, and the XGB classifier was implemented with the XGBoost package in R version 3.6.1. All performance metrics were calculated on the held‐out testing datasets. We generated confidence intervals (CIs) for performance metrics with epiR package of R software in training and testing datasets.

## 3 RESULTS

### 3.1 Patient characteristics

**FIGURE 1:**
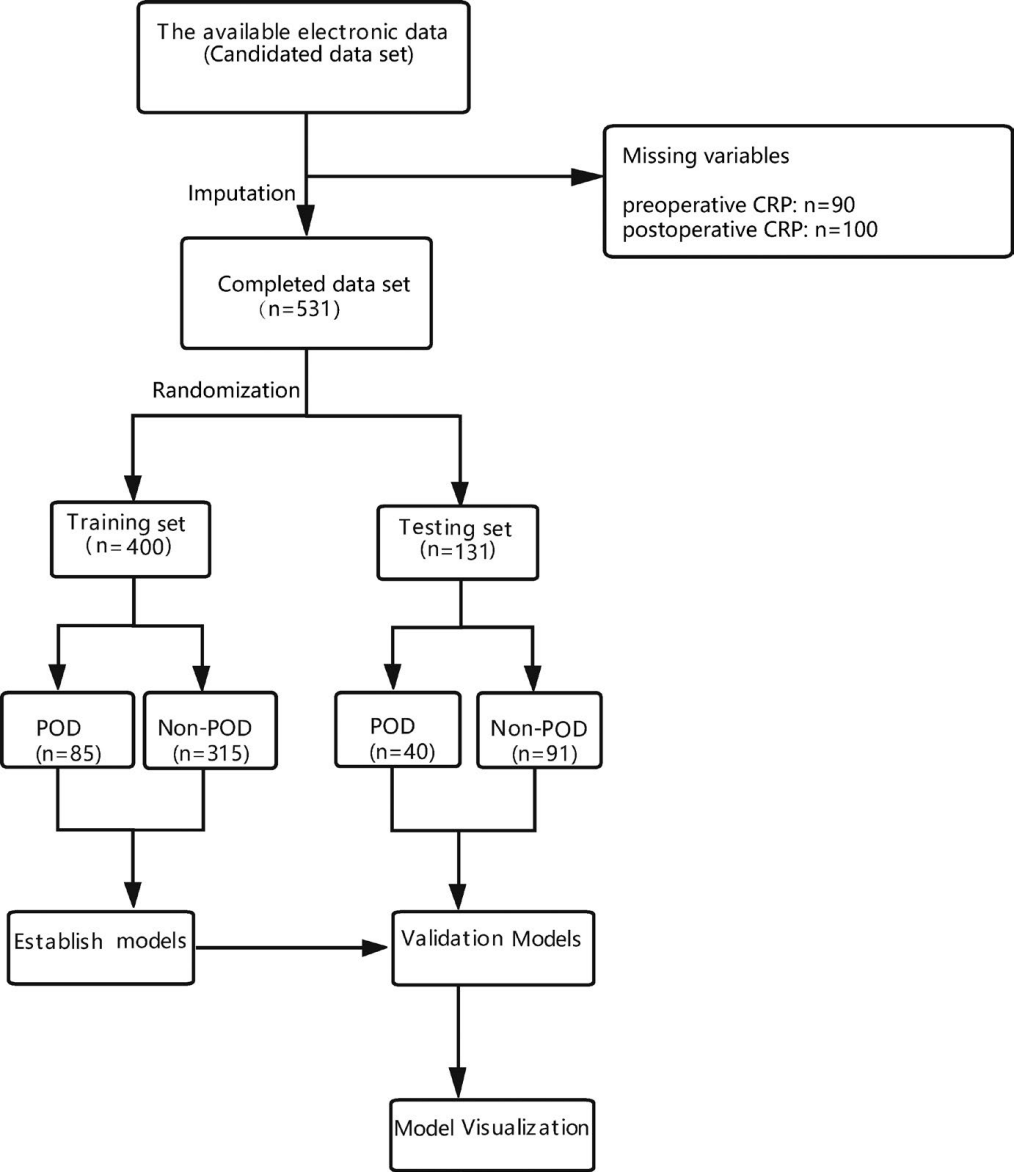
Patient recruitment flowchart

A total of 531 patients were included in this study. Among those screened, the incidence of POD was approximately 23.54%. The variables, including preoperative C‐reaction protein (CRP) and postoperative CRP, have missing values. Missing parts of these variables accounted for 16.9% and 18.8% of the total data, respectively. The missing data were imputed by random forest regression. The dataset (*n* = 531) is randomly divided into the training set and testing set at the ratio of 7:3. Four hundred patients formed a training dataset. One hundred thirty‐one patients formed testing datasets. The data collected from training datasets were used to assess important variables associated with POD and to establish the predictive models. Patients were divided into POD group (*n* = 125) and Non‐POD group (*n* = 406) according to whether or not delirium occurred within the first three days after surgery. The data collected from the testing dataset were aimed to validate predictive models. The patients' recruitment flowchart is shown in Figure 1. Detailed information on patient characteristics can be found in Table 1. There was no significant statistical difference between the features of patients in the training datasets and the testing datasets. The selection of the best parameter (lambda) in the LASSO model uses 10‐fold cross‐validation. Dotted vertical lines were drawn at the optimal values by using the minimum criteria and the 1 SE of the minimum criteria (the 1 − SE criteria).

**TABLE 1:** Comparison of demographic characteristics and perioperative and postoperative variables between the training dataset and the testing dataset

Property	Training dataset	Testing dataset	*p*
Patients, *n*	400	131	
Sex			0.324
Female, *n* (%)	165 (41.2%)	47 (35.9%)	
Male, *n* (%)	235 (58.8%)	84 (64.1%)	
Age (median ± IQR)	68.00 (65.00, 73.25)	68.00 (64.00, 72.0)	0.443
Height (median ± IQR)	165.00 (159.80, 170.00)	162.00 (158.00, 170.00)	0.247
Weight (median ± IQR)	62.50 (56.00, 70.00)	61.00 (55.00, 70.00)	0.483
BMI (median ± IQR)	23.80 (21.25, 25.90)	23.40 (21.47, 25.95)	0.867
Education degree, *n* (%)			0.945
Illiteracy	114 (28.5%)	40 (30.5%)	
Primary education	106 (26.5%)	34 (26.0%)	
Junior high school education	109 (27.3%)	31 (23.7%)	
High school education	53 (13.3%)	21 (16.0%)	
University degree	14 (3.5%)	4 (3.1%)	
University degree above	4 (1.0%)	1 (0.8%)	
ASA degree, *n* (%)			0.390
Ⅰ	12 (3.0%)	5 (3.8%)	
Ⅱ	324 (81.0%)	106 (80.9%)	
Ⅲ	64 (16.0%)	19 (14.5%)	
Ⅳ	0 (0.0%)	1 (0.8%)	
Smoking, *n* (%)			0.836
None	250 (62.5%)	80 (61.1%)	
Yes	150 (37.5%)	51 (38.9%)	
Alcohol, *n* (%)			0.026^*^
None	296 (74.0%)	83 (63.4%)	
Yes	104 (26.0%)	48 (36.6%)	
Hypertension, *n* (%)			0.672
None	265 (66.2%)	84 (64.1%)	
Yes	135 (33.8%)	47 (35.9%)	
Diabetes, *n* (%)			0.993
None	349 (87.3%)	115 (87.8%)	
Yes	51 (12.8%)	16 (12.2%)	
Hemoglobin (median ± IQR)	130.00 (118.00, 141.00)	131.00 (118.5, 144.0)	0.726
Albumin (median ± IQR)	42.15 (38.70, 45.00)	41.70 (38.0, 44.80)	0.408
ALT (median ± IQR)	16 (12, 24)	16 (11, 22.5)	0.240
AST (median ± IQR)	19 (15, 23.0)	18 (15, 21)	0.093
BUN (median ± IQR)	5.10 (4.19, 6.10)	5.20 (4.25, 6.65)	0.248
Cr (median ± IQR)	62.00 (54.00, 71.00)	64.00 (53.00, 71.5.00)	0.834
Blood volume (median ± IQR)	100 (100, 200)	100 (100, 250)	0.261
Urine volume (median ± IQR)	400 (300, 400)	400 (300, 500)	0.170
Crystalloid solution (median ± IQR)	1250 (1000, 1500)	1350 (1000, 1550)	0.298
Ethoxyl volume (median ± IQR)	500 (500, 500)	500 (500, 500)	0.668
Gelatin volume (median ± IQR)	0 (0, 0)	0 (0, 0)	0.517
Blood transfusion (median ± IQR)	0 (0, 0)	0 (0, 0)	0.087
Surgery time (median ± IQR)	161.00 (110.00, 225.00)	165.00 (120.00, 220.00)	0.875
Anesthesia duration (median ± IQR)	200.00 (150.00, 260.00)	190.00 (150.00, 252.50)	0.914
Extubation time (median ± IQR)	17.00 (10.00, 23.00)	12.00 (12.00, 24.50)	0.809
ICU admission, *n* (%)			0.493
None	333 (83.3%)	113 (86.5%)	
Yes	67 (16.8%)	18 (13.7%)	
MMSE (median ± IQR)	25.50 (23.00, 28.00)	26.00 (23.50, 28.00)	0.737
CCI (median ± IQR)	3.00 (2.00, 4.00)	3.00 (2.00, 4.00)	0.088
PSMS (median ± IQR)	6.00 (6.00, 6.00)	6.00 (6.00, 6.00)	0.334
IADL (median ± IQR)	8.00 (8.00, 8.00)	8.00 (8.00, 8.00)	0.648
QoR40 preoperative (median ± IQR)	195.00 (190.00, 198.00)	196.00 (190.00, 198.00)	0.770
PCA pump, *n* (%)			0.677
None	149 (37.2%)	46 (35.1%)	
Yes	251 (62.7%)	85 (64.9%)	
Nerve block, *n* (%)			1
None	286 (71.5%)	94 (71.8%)	
Yes	114 (28.5%)	37 (28.2%)	
Surgery type, *n* (%)			0.229
Thoracic operation	130 (32.5%)	44 (33.6%)	
Abdominal operation	183 (45.8%)	57 (43.5%)	
Urinary operation	68 (17%)	24 (18.3%)	
Orthopedic operation	19 (4.8%)	6 (4.6%)	
K+ (median ± IQR)	4.01 (3.75, 4.27)	4.01 (3.75, 4.27)	0.832
Glu (median ± IQR)	5.26 (4.81, 5.96)	5.26 (4.83, 5.91)	0.992
CRP preoperative (median ± IQR)	2.70 (1.10, 11.32)	3.60 (1.20, 10.70)	0.591
CRP postoperative (median ± IQR)	73.45 (42.90, 103.0)	76.80 (37.90, 124.00)	0.251
Cholesterol (median ± IQR)	4.59 (3.87, 5.13)	4.37 (3.79, 5.06)	0.392
Preoperative White blood cell count (median ± IQR)	5.90 (4.80, 7.20)	5.50 (4.80, 6.70)	0.394
Preoperative neutrophil count (median ± IQR)	3.63 (2.74, 4.72)	3.37 (2.81, 4.24)	0.538
Preoperative lymphocyte count (median ± IQR)	1.60 (1.20, 2.00)	1.50 (1.20, 1.90)	0.706
Postoperative White blood cell count (median ± IQR)	10.20 (8.40, 12.55)	10.10 (8.25, 12.20)	0.581
Postoperative neutrophil count (median ± IQR)	8.80 (7.02, 10.91)	8.34 (6.42, 10.86)	0.294
Postoperative lymphocyte count (median ± IQR)	0.90 (0.60, 1.20)	0.90 (0.70, 1.15)	0.701
Postoperative NLR (median ± IQR)	9.98 (6.54, 14.90)	9.66 (5.67, 14.12)	0.349
Preoperative NLR (median ± IQR)	2.27 (1.58, 3.26)	2.19 (1.72, 3.00)	0.934
Postoperative delirium, *n* (%)			0.040^*^
None	315 (78.7%)	91 (69.5%)	
Yes	85 (21.3%)	40 (30.5%)	

Abbreviations: ALT, alanine transaminase; ASA, American society of anesthesiologists; AST, glutamic oxalacetic transaminase; BMI, body mass index (kg/m^2^); BUN, blood urea nitrogen; CCI, Charlson comorbidity index; Cr, serum creatinine; CRP, C‐reactive protein; IADL, instrumental activities of daily living; MMSE, mini‐mental state examination score; NLR, neutrophil‐to‐lymphocyte ratio; PCA, postoperative analgesia pump; PSMS, physical self‐maintenance scale; QoR40, recovery quality rating scale.

**FIGURE 2:**
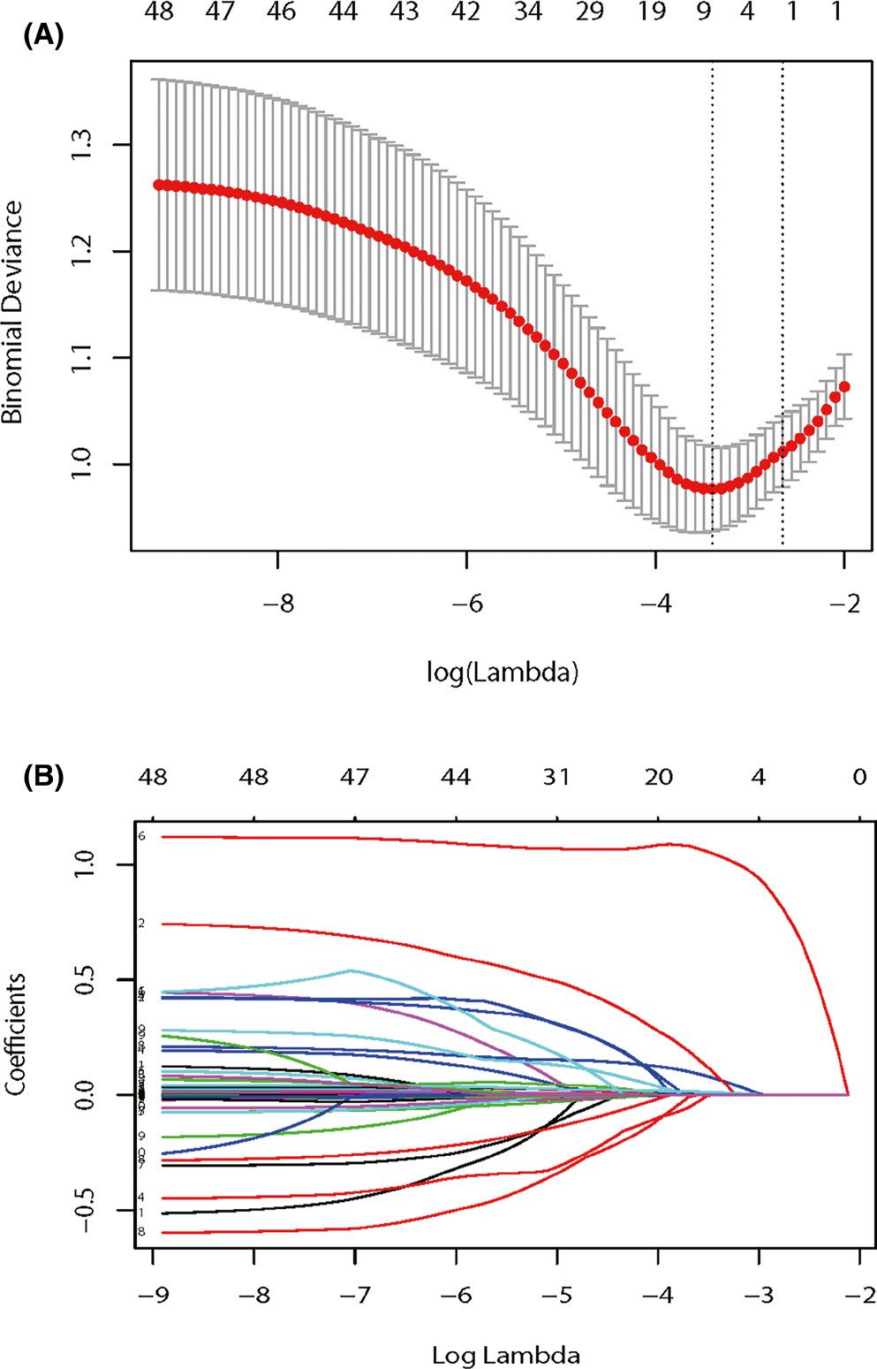
Demographic and clinical feature selection using the LASSO regression

A vertical line was drawn at the value selected using 10‐fold cross‐validation, where optimal lambda resulted in eight features with non‐zero coefficients (Figure 2A,B). We selected eight non‐zero characteristic variables in the LASSO regression results, including age, intraoperative blood loss, anesthesia duration, extubation time, ICU admission, MMSE score, CCI score, and postoperative NLR (Table 2).

**TABLE 2:** LASSO regression results of important variables related to POD (training dataset)

Variables	Coefficient	Lambda.min
Age	0.011537909	0.0345332
Intraoperative blood loss	0.0002223647	
Anesthesia duration	0.0017088601	
Extubation time	0.004272257	
ICU admission	0.951368637	
MMSE score	0.0066777804	
CCI	0.088073881	
Postoperative NLR	0.010530093	

Abbreviations: CCI, Charlson comorbidity index; MMSE, mini‐mental state examination score; NLR, neutrophil‐to‐lymphocyte ratio.

### 3.2 Model performance

We used four algorithms to build predictive models of POD, and the following values in the training datasets were found: LR classifier (AUC value = 73.99% (95%CI: 67.63%–80.35%), accuracy = 0.708 (95%CI: 0.660–0.752), precision = 0.701 (95%CI: 0.652–0.753), recall = 0.913 (95%CI: 0.872–0.942)); RF classifier algorithm (AUC value = 99.06% (95%CI: 97.74%–100%), accuracy = 0.993 (95%CI: 0.978–0.999), precision = 0.991 (95%CI: 0.982–1.000), recall = 1.0000 (95%CI: 0.982–1.000)); XGB classifier (AUC value = 89.77% (95%CI: 86.21%–93.32%), accuracy = 0.868 (95%CI: 0.8303–0.899), precision = 0.931 (95%CI: 0.892–0.953), recall = 0.911 (95%CI: 0.870–0.941)); SVM classifier (AUC value = 87.39% (95%CI: 82.84%–91.94%), accuracy = 0.913 (95%CI: 0.880–0.938), precision = 0.941 (95%CI: 0.910–0.964), recall = 0.951 (95%CI: 0.922–0.974)) (Table 3).

**TABLE 3:** Performance metrics for four models in training dataset

Model	Accuracy	F1 score	Precision	Recall	Specificity
LR	0.708 (0.660, 0.752)	0.494	0.701 (0.652, 0.753)	0.913 (0.872, 0.942)	0.382 (0.302, 0.464)
RF	0.993 (0.978, 0.999)	0.981	0.991 (0.982, 1.000)	1.000 (0.982, 1.000)	0.971 (0.912, 1.000)
XGB	0.868 (0.8303, 0.899)	0.654	0.931 (0.892, 0.953)	0.911 (0.870, 0.941)	0.682 (0.564, 0.782)
SVM	0.913 (0.880, 0.938)	0.785	0.941 (0.910, 0.964)	0.951 (0.922, 0.974)	0.763 (0.664, 0.851)

Accuracy = (TP + TN)/(TP + TN + FP + FN). Precision = TP/(TP + FP). Recall = TP/(TP + FN). Specificity = TN/(TN + FP). F1 score = 2/([1/Recall] + [1/Precision]). FN, false negatives; FP, false positives; TN, true negatives; TP, true positives.

**FIGURE 3:**
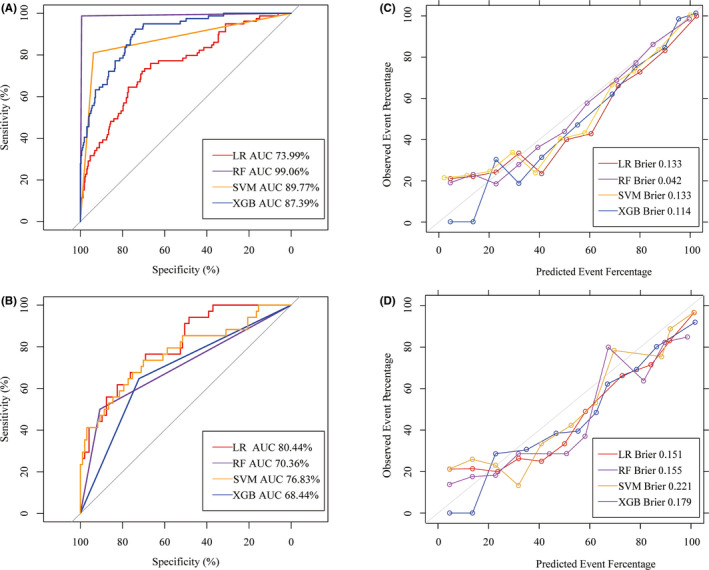
ROC of models and calibration plot in training dataset and testing dataset (A and C represented training dataset. B and D represented testing dataset)

For the testing dataset, the following values in the test group were found: LR classifier (AUC value = 80.44% (95%CI: 72.24%–88.64%), accuracy = 0.687 (95%CI: 0.600–0.765), precision = 0.661 (95%CI: 0.563–0.754), recall = 0.891 (95%CI: 0.791–0.950); RF classifier (AUC value = 70.36% (95%CI: 61.35%–79.37%), accuracy = 0.801 (95%CI: 0.723–0.866), precision = 0.912 (95%CI: 0.832–0.962), recall = 0.842 (95%CI: 0.751–0.904); XGB classifier (AUC value = 76.83% (95%CI: 66.77%–86.89%), accuracy = 0.779 (95%CI: 0.698–0.847), precision = 0.881 (95%CI: 0.792–0.930), recall = 0.832 (95%CI: 0.753–0.901); SVM classifier (AUC value = 68.44% (95%CI: 59.13%–77.74%), accuracy = 0.702 (95%CI: 0.616–0.779), precision = 0.723 (95%CI: 0.622–0.812), recall = 0.852 (95%CI: 0.763–0.924)) (Table 4). The ROC of models in testing dataset and training dataset is shown in Figure 3A,B, and the AUROC for each model is shown in Table 5.

**TABLE 4:** Performance metrics for four models in testing dataset

Model	Accuracy	F1 score	Precision	Recall	Specificity
LR	0.687 (0.600, 0.765)	0.559	0.661 (0.563, 0.754)	0.891 (0.791, 0.950)	0.442 (0.311, 0.583)
RF	0.801 (0.723, 0.866)	0.567	0.912 (0.832, 0.962)	0.842 (0.751, 0.904)	0.651 (0.442, 0.833)
XGB	0.779 (0.698, 0.847)	0.539	0.881 (0.792, 0.930)	0.832 (0.753, 0.901)	0.592 (0.390, 0.761)
SVM	0.702 (0.616, 0.779)	0.530	0.723 (0.622, 0.812)	0.852 (0.763, 0.924)	0.452 (0.311, 0.602)

Accuracy = (TP + TN)/(TP + TN + FP + FN). Precision = TP/(TP + FP). Recall = TP/(TP + FN). Specificity = TN/(TN + FP). F1 score = 2/([1/Recall] + [1/Precision]). FN, false negatives; FP, false positives; TN, true negatives; TP, true positives.

**TABLE 5:** Delirium prediction performance using AUROC

Model	Training sets AUC (95%CI)	Testing sets AUC (95%CI)
LR	73.99% (67.63%−80.35%)	80.44% (72.24%−88.64%)
RF	99.06% (97.74%−100%)	70.36% (61.35%−79.37%)
XGB	89.77% (86.21%−93.32%)	76.83% (66.77%−86.89%)
SVM	87.39% (82.84%−91.94%)	68.44% (59.13%−77.74%)

Abbreviations: LR, logistic regression; RF, random forest; SVM, support vector machine; XGB, extreme gradient boosting.

The LR achieved much lower (better) Brier scores compared with the other models. Calibration plots of four models in the training dataset and testing dataset are shown in Figure 3C,D. The curve at 45° between the *X*‐axis and the *Y*‐axis indicates good consistency of the model.

**FIGURE 4:**
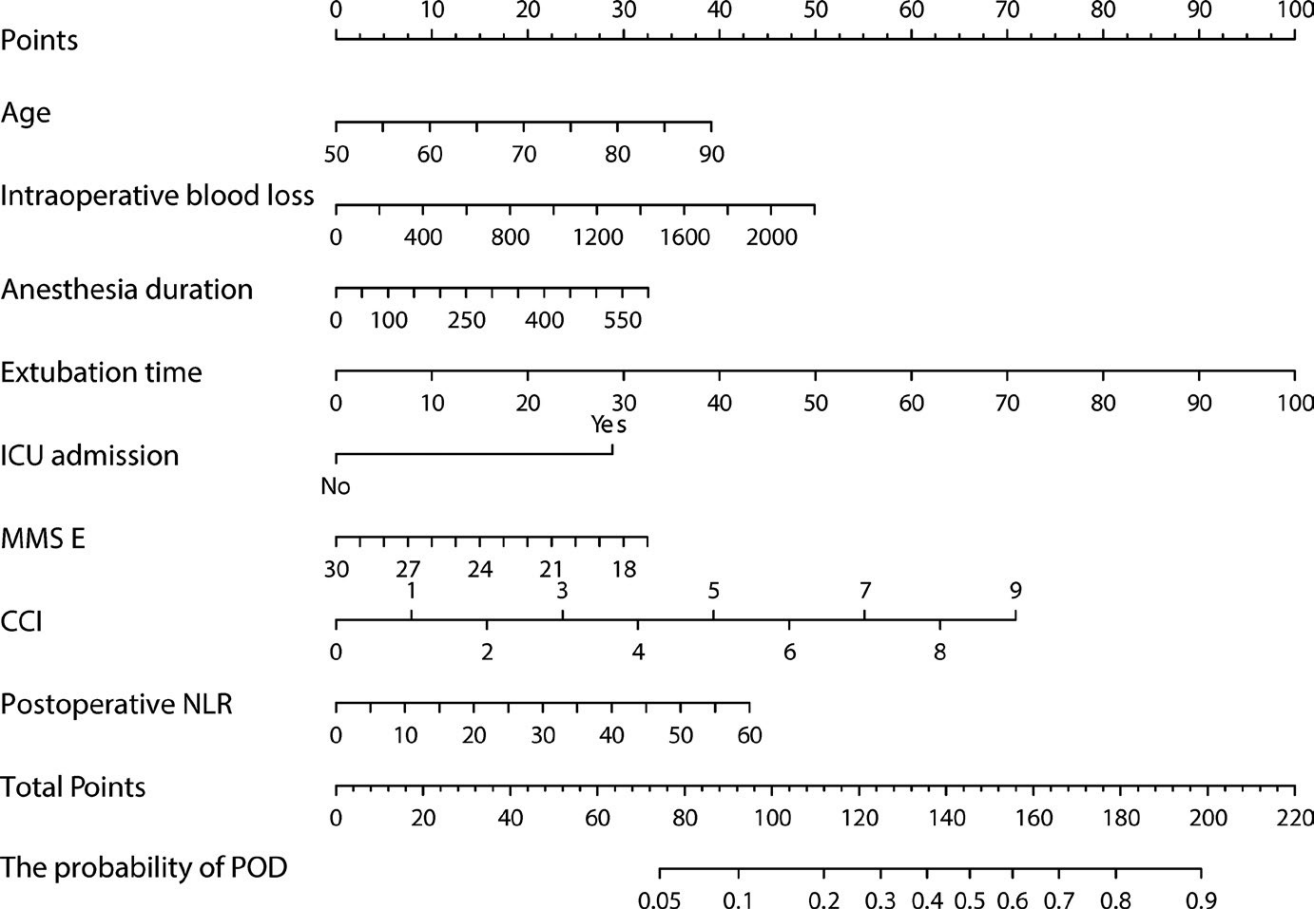
Nomogram for estimation of POD

Finally, the LR model is transformed into a nomogram to understand and use the model (Figure 4). The two‐class prediction outcome is generated based on the optimal cutoff value of the optimal model. Comparing the prediction outcome with the actual occurrence of delirium, the optimal model has shown that the prediction outcome has good performance. The optimal cutoff value of the LR model corresponds to the optimal score of the nomogram. The optimal score of the nomogram was determined to be 109 according to the optimal cutoff value of the LR model. If the sum of the scores corresponding to each entry in the nomogram is greater than 109 points, then the patients who underwent surgery have a higher risk of developing POD. At this moment, nursing staff and doctors should pay attention to the situation of patients. Table 6 presents that these variables including age (OR = 1.054, 95%CI: 1.017~1.093), extubation time (OR = 1.027, 95%CI: 1.012~1.044), ICU admission (OR = 2.238, 95%CI: 1.313~3.793), MMSE (OR = 0.929, 95%CI: 0.876~0.984), CCI (OR = 1.197, 95%CI: 1.038~1.384), and postoperative NLR (OR = 1.029, 95%CI: 1.002~1.057) were independent risk factors for POD in this study (Table 6).

**TABLE 6:** Multivariate logistic regression analysis results in training set

Variables	β coefficient	OR (95%CI)	*p*‐value
Age	0.053	1.054 (1.017~1.093)	0.003^*^
Intraoperative blood loss	0.001	1.000 (0.999~1.001)	0.149
Anesthesia duration	0.002	1.002 (0.999~1.005)	0.076
Extubation time	0.027	1.027 (1.012~1.044)	0.006^*^
ICU admission	0.806	2.238 (1.313~3.793)	0.002^*^
MMSE	−0.074	0.929 (0.876~0.984)	0.012^*^
CCI	0.179	1.197 (1.038~1.384)	0.014^*^
Postoperative NLR	0.028	1.029 (1.002~1.057)	0.034^*^

*p < 0.05

Abbreviations: CCI, Charlson comorbidity index; MMSE, mini‐mental state examination score; NLR, neutrophil‐to‐lymphocyte ratio.

## 4 DISCUSSION

The accumulation of multiple risk factors is critical for the occurrence of POD, and there is currently no single treatment to prevent the occurrence of POD. The combination of non‐drug therapy and drug therapy is one of the best methods to treat POD. POD has been reported to occur in 10% to 70% of all elderly patients,^1^, ^2^, ^3^ causing increased mortality, prolonged hospital stays, reduced functional abilities,^20^, ^21^ long‐term cognitive dysfunction,^22^ and even dementia.^15^, ^23^ Therefore, the prevention and treatment of POD is a clinical problem that needs to be solved. In many clinical studies on POD, researchers have tried to find powerful biomarkers that can accurately predict POD, such as S100β protein,^24^ neuron‐specific enolase (NSE),^25^ tau protein,^26^ and inflammatory mediators.^27^ Researchers are also trying to find better ways to reduce the occurrence of POD. Although these biomarkers have a relatively high ability to predict POD, they cannot be popularized clinically because of the complexity and high cost of sampling. They are always used to explore scientific questions in clinical trials. Therefore, the emergence of disease prediction models may provide a solution for the prevention of POD. Neuroinflammation and the oxidative stress response may be involved in the pathophysiological process of POD.^5^, ^6^ Inflammatory markers investigated in scientific studies have been associated with delirium.^7^, ^8^, ^9^ To further increase the general applicability of the model, this study included easily available laboratory test items, including some inflammatory mediators, such as CRP and the NLR variables.

There are many ways to build a POD prediction model, but many mathematical terms are always involved.^12^, ^28^ This is not conducive to the understanding and use of a model by medical staff. At the same time, many disease prediction models are transformed into certain formulas, limiting the availability of prediction models.^11^, ^12^ Therefore, the model established in this study was transformed into a nomogram to increase the availability of the model further.

In this study, we established a predictive model and incorporated the following eight variables into its construction: age, intraoperative blood loss, anesthesia duration, extubation time, ICU admission, MMSE score, CCI score, and postoperative NLR. The optimal predictive model was represented by a nomogram. It is a new concept to use a nomogram to estimate the risk of POD. The LR model performed well, with AUCs of 73.99% and 80.44% in the training and testing datasets, respectively. The calibrations of the models were compared quantitatively using Brier scores. The calibration of the LR model showed good agreement between the prediction outcome and the actual observed outcome. For the application of this model, the sum of the scores corresponding to each entry in the nomogram was more significant than 109 points, and patients who underwent surgery had a higher risk of developing POD. Based on this predictive model, the nomogram can be used as a tool to screen out patients with a high risk of POD. Thus, targeted interventions can be made for high‐risk patients.

Finally, eight variables were included in the multivariate logistic regression analysis. We found that age, extubation time, ICU admission, MMSE score, CCI score, and postoperative NLR were independent risk factors for POD. Advanced age is known to be the most relevant risk factor for POD, and some basic systemic diseases before surgery may also increase the incidence of POD.^29^, ^30^ Entering the ICU after surgery may also increase the incidence of POD, which may be related to the ICU environment, long‐term mechanical ventilation, and the severity of the patient's disease.^31^ The MMSE assesses cognitive function in patients and is associated with POD.^32^ These findings are consistent with our study. Extubation time is related to residual anesthetic drugs at the end of the anesthesia maintenance period and the patient's disease state before surgery. This study also confirmed that extubation time is a risk factor for POD. The postoperative NLR is also related to POD, but CRP variable was excluded when we screened for important features in this study.

On the one hand, we infer that the NLR, a parameter derived from different white blood cell counts, is a synthesized marker of both inflammation and oxidative stress and a stronger inflammatory factor than CRP variable.^7^, ^8^ On the other hand, CRP variable has a certain amount of missing data. Although we imputed missing data, this could still affect the screening of important features. Considering the above two aspects, the missing data could lead to the exclusion of the CRP variable and the NLR inclusion. However, two variables, intraoperative blood loss and anesthesia duration, were excluded by multivariate logistic regression. These were inconsistent with some previous research findings.^33^, ^34^ Considering that these two variables may be potential risk factors for POD and the principle of the minimum Akaike information criterion (AIC) and the maximum AUROC of the prediction model, we finally included these two variables in the established models. Predictive models built using independent risk factors could ignore this principle and fail to achieve the best model.

However, there are still several limitations of this study. First, this is a small sample study, and the predictive model requires a larger sample for verification. Second, the interpolation of missing data is a complex problem because data were considered to be randomly missing. In fact, there is a large field of research that builds optimal imputation algorithms, and suboptimal imputation algorithms will decrease the performance of the predictive model. This may be a possible reason why the performance of our model is lower than that of the previous models.^35^ However, our choice to use imputation algorithms,^36^, ^37^ while not optimal, was better than using mean imputation. Third, the data in this study came from a single large academic medical center. Thus, this model may not have similar effects when used in other medical institutions. Most likely, the model will need to be recalibrated when used by another institution. The exact weights of the features may change through such recalibration. Finally, this model requires an independent dataset to test the extrapolation and generalization of the model. We hope to collect enough external validation datasets to improve this model in the future further.

The benefits of machine‐learning technology are large, especially in the medical industry. For example, using machine‐learning technology to establish disease prediction and risk assessment models can help clinicians better identify the factors that truly drive the occurrence and development of diseases.

## 5 CONCLUSIONS

We developed four different POD prediction models and calibrated them with Brier Score to select the model with the best performance. We believe that the model is an important tool that should be utilized to screen out the high‐risk group of POD.

## DISCLOSURE STATEMENT

The authors declare that they have no competing interest.

## AUTHORS' CONTRIBUTIONS

Jun‐Li Cao designed the study, critically reviewed the manuscript, approves the final version, and is accountable for the work. Xiao‐Yi Hu, He Liu, and Yuan Han designed the study, conducted the study, collected the data, prepared the manuscript, critically reviewed the manuscript, approved the final version, and are accountable for the work. Xing Gao, Yang Zhou, and Jian Zhou helped conduct the study and collected the data. Hui‐Lian Guan and Xun Sun analyzed and interpreted the data. Xue Zhao and Qiu Zhao helped prepare the manuscript and critically reviewed the manuscript.

## Data Availability

The data that support the findings of this study are available from the corresponding author upon reasonable request.
